# Immunotherapy Study on Non-small-Cell Lung Cancer (NSCLC) Combined with Cytotoxic T Cells and miRNA34a

**DOI:** 10.1021/acs.molpharmaceut.3c01040

**Published:** 2024-01-31

**Authors:** Richa Pandey, Chien-Chih Chiu, Li-Fang Wang

**Affiliations:** † Department of Medicinal and Applied Chemistry, Kaohsiung Medical University, No. 100 Shih-Chuan first Road, Kaohsiung 80708, Taiwan; ‡ Department of Biotechnology, Kaohsiung Medical University, No. 100 Shih-Chuan first Road, Kaohsiung 80708, Taiwan; § Department of Medical Research, Kaohsiung Medical University Hospital, No.100 Tzyou first Road, Kaohsiung 80708, Taiwan; ∥ Institute of Medical Science and Technology, National Sun Yat-Sen University, No.70 Lien-Hai Road, Kaohsiung 804201, Taiwan

**Keywords:** Jurkat T cells, miRNA34a, iron oxide nanoparticles, PD-L1, immunotherapy

## Abstract

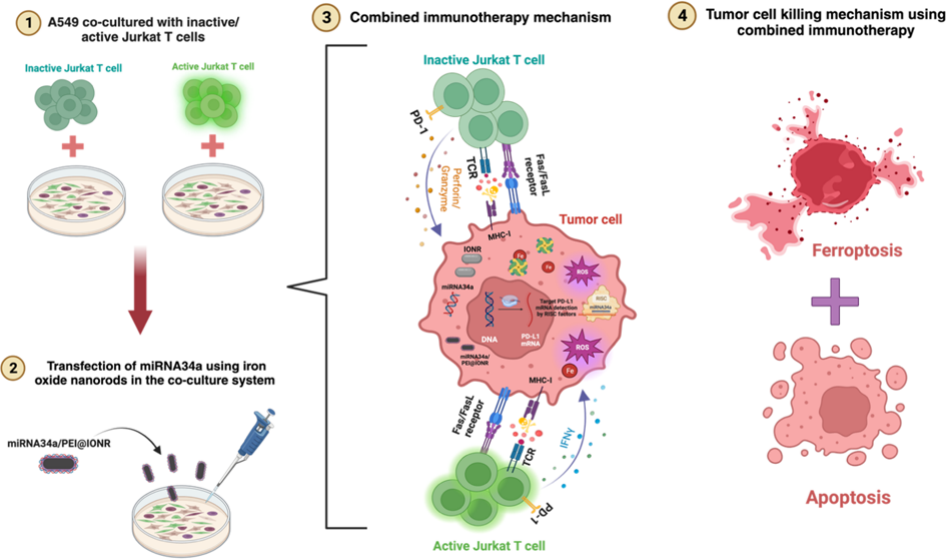

Immunotherapy has emerged as a promising approach for cancer treatment, and the use of microRNAs (miRNAs) as therapeutic agents has gained significant attention. In this study, we investigated the effectiveness of immunotherapy utilizing miRNA34a and Jurkat T cells in inducing cell death in non-small-cell lung cancer cells, specifically A549 cells. Moreover, we explored the impact of Jurkat T cell activation and miRNA34a delivery using iron oxide nanorods (IONRs) on the killing of cancer cells. A549 cells were cocultured with both activated and inactivated Jurkat T cells, both before and after the delivery of miRNA34a. Surprisingly, our results revealed that even inactive Jurkat T cells were capable of inducing cell death in cancer cells. This unexpected observation suggested the presence of alternative mechanisms by which Jurkat T cells can exert cytotoxic effects on cancer cells. We stimulated Jurkat T cells using anti-CD3/CD28 and analyzed their efficacy in killing A549 compared to that of the inactive Jurkat T cells in conjunction with miRNA34a. Our findings indicated that the activation of Jurkat T cells significantly enhanced their cytotoxic potential against cancer cells compared to their inactive counterparts. The combined treatment of A549 cells with activated Jurkat T cells and miRNA34a demonstrated the highest level of cancer cell death, suggesting a synergistic effect between Jurkat T cell activation and miRNA therapy. Besides the apoptosis mechanism for the Jurkat T cells’ cytotoxic effects on A549 cells, we furthermore investigated the ferroptosis pathway, which was found to have an impact on the cancer cell killing due to the presence of miRNA34a and IONRs as the delivery agent inside the cancer cells.

## Introduction

Cancer continues to be a significant global health challenge, with millions of lives affected by this complex and heterogeneous disease. Conventional treatment modalities such as surgery, chemotherapy, and radiation therapy have made substantial progress, but they often come with limitations such as systemic toxicity, drug resistance, and damage to healthy tissues.^1,2^ In recent years, the field of cancer immunotherapy has emerged as a revolutionary approach that harnesses the power of the immune system to specifically target and eliminate cancer cells. Among the various strategies employed, Jurkat T cells have gained significant attention for their potential in cancer immunotherapy.^1,3^ In recent years, the integration of nanotechnology into immunotherapy has opened up new avenues for precise and targeted cancer treatment.^4^ Within this field, the integration of nanoparticles and Jurkat T cell immunotherapy has shown great promise as a novel and effective strategy for cancer treatment.^5^ Nanoparticles can be engineered to carry specific payloads, such as chemotherapeutic drugs, genes, or immunomodulatory molecules, they can be surface modified or/and manipulated for cancer theranostics approach, such as real-time guidance in single, dual, or multiplex imaging modalities, aiding or assisting in cancer treatment and enhance their therapeutic efficacy.^5−7^

Jurkat T cells, a well-established human T-cell leukemia cell line, possess inherent antitumor properties and can be genetically modified to augment their effectiveness in cancer immunotherapy.^8^ The cells have the ability to recognize and attack cancer cells, offering a unique approach to specifically target tumor sites. Jurkat T cells employ a multifaceted approach to eliminate cancer cells, combining direct cytotoxicity, secretion of cytokines and chemokines, antibody-dependent cell-mediated cytotoxicity, and antibody-dependent cell-mediated phagocytosis.^9^ By introducing genetic modifications, Jurkat T cells can be engineered to express chimeric antigen receptors or T-cell receptors (CAR-T) that recognize tumor-specific antigens, further enhancing their tumor-killing capabilities.^10^ Jurkat T cells provide a valuable tool for evaluating the functionality and effectiveness of these CARs in a controlled laboratory setting, which target multiple antigens simultaneously.^11^ Modification of Jurkat T cells into CAR-Jurkat T therapy is a revolutionary approach that involves nanoparticle-based CAR immune therapy. When nanoparticles fabricated with a vaccine or genetic materials are introduced into the cells, the internalization of NPs takes place through the interaction between the surface antigen of the targeted T cells and the surface antibody of the NPs within the living organism. This process enables T cells to express CARs, which further leads to the engagement of CAR-T cells with the targeted cancer cells, leading to their activation, rapid cell division, and the induction of cytotoxic effects on the intended tumor.^12,13^ Several studies highlighted the cell therapy by using titanium oxide (TiO_2_), which forms a coating on individual Jurkat cells.^14^ Due to this TiO_2_, there will be no loss in cell viability, and Jurkat T cells can produce higher cytokines in cancer immunotherapy.^14^ Schneck et al. have shown the use of Jurkat T cells in adoptive T cell therapy by using iron-dextran nanoparticles coated with MHC II and costimulatory proteins to enhance CD8^+^ T cell cells activity, leading to an increased cytokine production and memory formation to kill cancer cells more efficiently.^15^ Many combined blockade therapy approaches are also being followed, such as PD-1/PD-L1 blockage or triple blockade of CTLA-4, PD-L1, and TIM3 receptors, in order to enhance the functions of T cells.^16,17^ Researchers are also exploring combinations of CAR-T cell therapy with other treatments such as immune checkpoint inhibitors and targeted therapies. This approach can enhance the effectiveness of CAR-T therapy and potentially extend its use to a broader range of cancer types. For example, combining CAR-T therapy with PD-1 inhibitors and cytokines such as IL-4 has shown promise in treating solid tumors.^18^ Jurkat T cells are also being used in drug screening to evaluate the efficacy of various compounds and potential immunotherapeutic agents.^19^ This aids in the identification of novel drugs that can enhance the anticancer immune response and improve treatment outcomes.^20^ Jurkat T cells are cornerstones in cancer immunotherapy research and development. Their utility in modeling T cell behavior, evaluating CAR-T cell therapies, and testing novel drugs is invaluable for advancing the field and ultimately improving the treatment options available for cancer patients. However, it is essential to note that T cell therapy is still an evolving field, and ongoing research and clinical trials will continue to shape its future.

We have demonstrated in our previous study that miRNA34a carries the potential to silence programmed cell death ligand 1 (PD-L1) genes in triple-negative breast cancer and non-small-cell lung cancer (NSCLC) after introducing inside the cells using a nonviral delivery vector like iron oxide nanorods (IONRs).^21^ This immunotherapy approach using magnetic nanoparticles to deliver miRNA34a proved to be one of the efficient therapeutic approaches to kill cancer cells. However, in the tumor microenvironment, cancer cells often exploit immune checkpoint pathways, such as PD-L1 pathway, to evade immune detection and attack.^22^ This immune evasion mechanism hampers the efficacy of T cells in eliminating cancer cells. To overcome this challenge, researchers have utilized miRNA34a to disable the immunosuppressive signals mediated by this pathway, thereby enhancing the T cells ability to recognize and destroy cancer cells.^23^ Considering immunotherapy, ferroptosis can also be triggered either by directly targeting the iron metabolism of cancer cells or by modulating the immune response to enhance the sensitivity of tumor cells to ferroptosis. Strategies such as administration of ferroptosis-inducing agents, including small molecules or nanoparticles, or genetic manipulation of key regulatory proteins involved in ferroptosis, have shown potential in enhancing the antitumor immune response.^24^ Ferroptosis induction in immunotherapy offers a new avenue for selectively eliminating cancer cells while minimizing damage to healthy tissues, making it an exciting area of research with promising therapeutic implications. Additionally, to enhance the delivery of miRNA34a into cancer cells, IONRs have been utilized as a magnetofection agent. The use of IONRs as a gene delivery agent has previously been successfully established as safe, minimizing cytotoxicity concerns associated with other transfection reagents.^21^

Immunotherapy has emerged as a promising approach in the treatment of various cancers, including NSCLC. It is a prevalent type of lung cancer with limited treatment options, and innovative strategies are continuously being explored to improve patient outcomes.^1,25^ Jurkat T cells are activated when they recognize specific antigens on the surfaces of cancer cells. These antigens can be proteins or peptides derived from cancer-specific or tumor-associated antigens.^26^ Some of the activated Jurkat T cells differentiate into cytotoxic T lymphocytes (CTLs), which are specialized in killing target cells, including cancer cells.^26^ CTLs release cytotoxic molecules, such as perforin and granzymes, to attack cancer cells. Perforin creates pores in the target cell’s membrane, allowing granzymes to enter, while granzymes initiate apoptosis in the cancer cell by cleaving specific proteins and activating caspases.^27^ Another mechanism used by T cells involves the Fas receptor and the Fas ligand (FasL). The Fas receptor on cancer cells interacts with the FasL on T cells, triggering apoptosis in cancer cells.^28^ Moreover, T cells can release cytokines, such as interferon-γ (INF-γ) and tumor necrosis factor, which stimulate immune responses and further enhance the body’s ability to combat cancer.^29^

**Figure 1:**
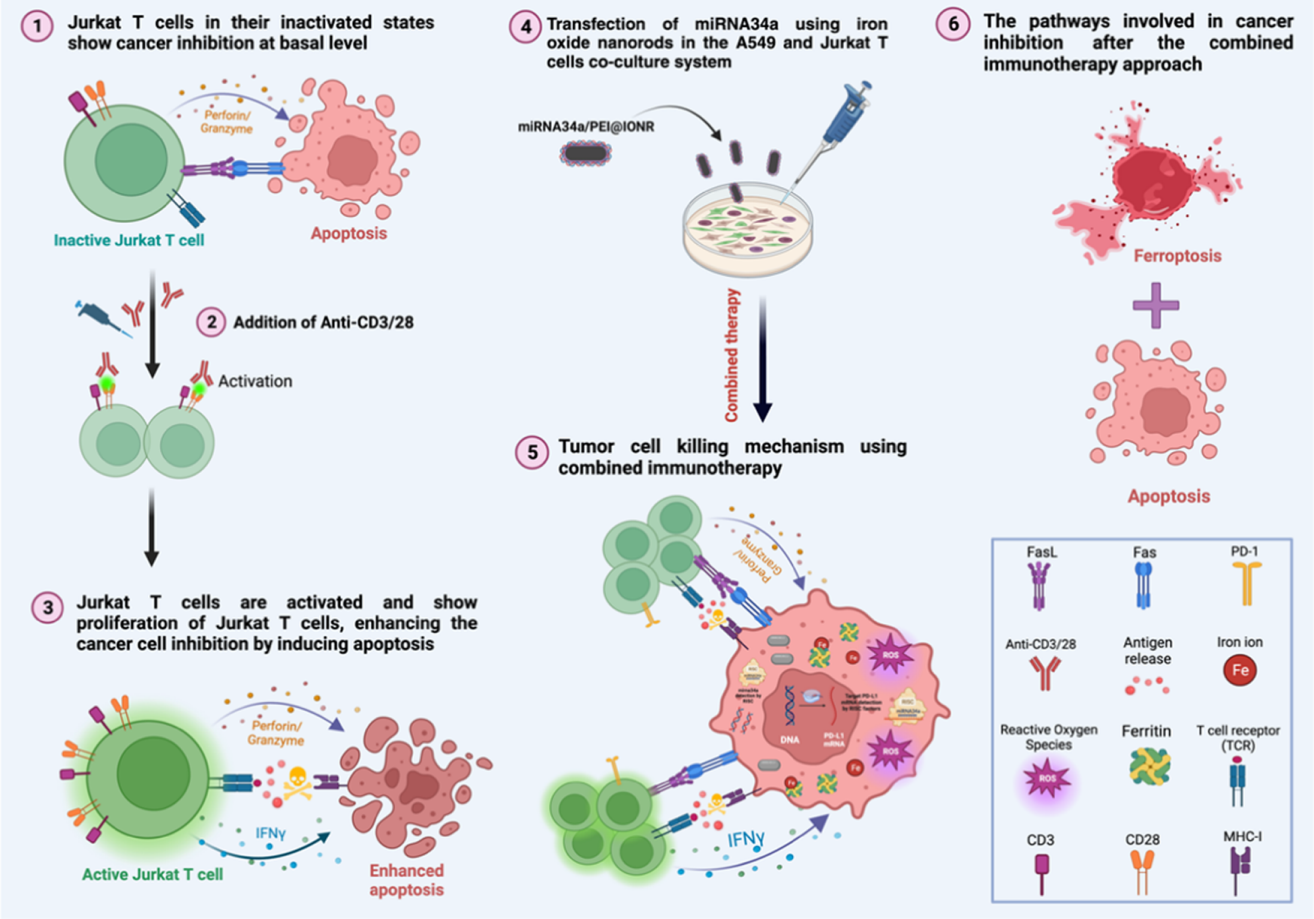
Illustration of the experimental setup and outcomes of the study. The cytotoxic effects of inactive and active Jurkat T cells on tumor cells in the presence of miRNA34a delivery using iron oxide nanorods. The figure provides a graphical representation of the observed cancer cell death induction by apoptosis and the impact of iron oxide nanorods along with miRNA34a and Jurkat T cells to promote ferroptosis along with apoptosis. The illustration was made using BioRender software.

In this study, we sought to elucidate the impact of inactive and active Jurkat T cell and miRNA34a transfection in a combined therapy approach on the immune response against NSCLC, as illustrated in Figure 1. The inactive Jurkat T cells were stimulated using anti-CD3/CD28, and a comparative study was carried out between inactive and active Jurkat T cells on cancer cell killing. The impact of miRNA34a is also studied when transfected inside cancer cells and along with inactive and active Jurkat T cells. CD8+ T cells express PD-1 receptors, while cancer cells carry PD-L1 receptors on their surfaces.^26^ The combination of both receptors leads to the weakening of the T cell immune functions against cancer cells, thereby leading to the tumor escape. By delivering miRNA34a, we aimed to suppress the activity of PD-L1 surface markers on the cancer cells, thereby allowing T cells to gain strength and attack the cancer cells by various cytotoxic mechanisms.^30^ Initially, we had cocultured inactive Jurkat T cells and A549 cells, subsequently stimulated Jurkat T cells to their active states using anti-CD3/CD28, and cocultured active Jurkat T cells with A549 cells. We anticipated that the cancer cell death will be comparatively higher by the active Jurkat T cells than by the inactive Jurkat T cells in the presence or absence of miRNA34a. The possible killing mechanisms could vary, such as ferroptosis, apoptosis, as well as necrosis, in the coculture experiments before and after the activation of Jurkat T cells and miRNA34a-transfected NSCLC cells. These findings can contribute to the development of novel immunotherapeutic strategies for NSCLC treatment, potentially improving patient outcomes in the future.

## Materials and Methods

### Plasmid and Cell Lines

A lentiviral vector-based GFP tagged hsa-miR-34a-1 pre-microRNA construct was purchased from System Biosciences. Plasmid DNA (pDNA) was amplified in chemically competent *Escherichia coli* strain DH5α (Yeastern Biotech, Taipei, Taiwan) and purified using a Geneaid Plasmid Maxi Kit (New Taipei City, Taiwan). The purity of pDNA was checked by the absorbance ratio at OD260/OD280 and by distinctive bands of DNA fragments at corresponding base pairs in gel electrophoresis after restriction enzyme treatments. A549 and Jurkat T lymphocyte cell lines were obtained from ATCC and maintained in RPMI-1640 medium, supplemented with 10% fetal bovine serum (FBS) and 100 μg/mL penicillin–streptomycin at 37 °C and 5% CO_2_.

### Reagents

3-(4,5-Dimethyl-thiazol-2yl)-2,5-diphenyl-tetrazolium bromide (MTT) was purchased from MP Biomedicals (Eschwege, Germany). Phosphate-buffered saline (PBS), RMPI-1640, Dulbecco’s modified Eagle’s medium (DMEM), Minimum Essential Medium (MEM), and trypsin–EDTA (Ethylenediaminetetraacetic) were purchased from Invitrogen (Carlsbad, CA). PolyMag was acquired from Chemicell GmbH (Berlin, Germany). 3-Zol (Trizol) reagent was purchased from Cyrusbioscience (Taipei, Taiwan). A BCA protein assay kit was purchased from Thermo Fisher Scientific Inc. (Rockford, IL). T cell TransAct human anti-CD3/CD28 reagents were purchased from Miltenyi Biotec. (Bergisch Gladbach, Germany).

### Coculture of Tumor Cells with Jurkat Cells and Luciferase Assay

For the coculture with activated and inactivated Jurkat T cells, 1 × 10^5^ A549 luciferase (Luc) cells were seeded in 6-well plates, and inactivated Jurkat T cells were added to the wells with increasing cell numbers ranging of 5 × 10^2^ (T1), 5 × 10^3^ (T2), 5 × 10^4^ (T3), and 5 × 10^5^ (T4). After 48 h of coculture, cells were harvested carefully, and cell lysates were prepared to check the cell viability by detecting the luciferase activity of the A549 luciferase cells. For sample preparations in general, to harvest the cells in a coculture condition, the coculture medium containing nonadherent Jurkat T cells and adherent A549 cells was aspirated carefully. The adherent A549 cells and remaining Jurkat T cells were washed twice gently with PBS to remove the residual culture medium. The PBS was aspirated after each wash, and 300–500 μL mL of RIPA buffer was added to the coculture and incubated for 5 min at room temperature. After 5 min, the cocultured cells were collected by scrapping thoroughly using a sterilized scrapper. Luciferase activity was measured by treating the cell lysates with luciferin buffer, and the cell luminescence was measured using the Luciferase system FLX800. Further, inactivated Jurkat T cells were treated with T cell TransACT anti-CD3/CD28 reagents, and the cell activator was added as per the manufacturer’s protocol. The same Luc activity of A549 cells cocultured with activated Jurkat T cells was assayed as aforementioned.

### Flow Cytometry

Two types of culture systems were established: tumor cells cocultured with inactive Jurkat cells and tumor cells cocultured with active Jurkat cells. A549 (1 × 10^6^) cells were seeded in 6-well plates, and after 24 h of incubation, Jurkat T cells in variable cell numbers of 5 × 10^3^, 5 × 10^4^, 5 × 10^5^, and 5 × 10^6^ were added in the wells with the tumor cells. Active or inactive Jurkat T cells were also cultured alone with a cell number of 5 × 10^4^. After 48 h of incubation, the coculture medium containing nonadherent Jurkat T cells was carefully aspirated. The adherent A549 cells and remaining Jurkat T cells were washed twice gently with 1× PBS to remove the residual culture medium. The PBS was aspirated after each wash, and 1 mL of 1× trypsin was added and incubated for 5 min at 37 °C. The cell pellet was collected by centrifugation at 1000 rpm for 5 min. The cell pellet was stained with Annexin V-fluorescein isothiocyanate (FITC) and propidium iodide (PI) for 15 min and further detected by using a flow cytometer (Beckman Coulter) and analyzed by using WinMDI software.

For the detection of CD3/CD28 expression in Jurkat T cells, 1 × 10^6^ A549 cells were seeded in 6-well plates. The active or inactive Jurkat T cells were cocultured with 1 × 10^6^ A549 cells after 24 h. Also, in one treatment, the A549 cells were transfected with miRNA34a and cocultured with active or inactive Jurkat T cells. After such treatments of A549 cells with active or inactive Jurkat T cells coculture and miRNA34a transfection, the cell samples were incubated for 72 h and collected by trypsinization and centrifugation at 1000 rpm for 5 min. The cell pellet was incubated with primary antibodies CD3 and CD28 for 1 h and washed with 1X PBS and further incubated with secondary antibodies for 30 min. Lastly, the cell pellet was washed with 1× PBS and further subjected to the detection of CD3/CD28 expression of Jurkat T cells by using flow cytometry and analyzed by using WinMDI software.

### Western Blot

A549 cells (7 × 10^4^) were cocultured with 5 × 10^3^ active or inactive Jurkat T cells in 12-well plates. The cocultures were treated with and without miRNA34a and incubated for 48 h. After 48 h, the cells were harvested by aspirating the coculture medium very gently and carefully. The adherent A549 cells and Jurkat T cells were washed twice gently with PBS to remove the residual culture medium, and the PBS was aspirated carefully after each wash. Then, 300–500 μL mL of protein lysis buffer, which is RIPA buffer, was added to the coculture and incubated for 5 min at room temperature. The cells were lysed by protein lysis buffer and collected by scrapping the cell culture plate using a scraper to take out the maximum cell lysate. The protein concentration of the cell lysate was determined using a BCA protein assay kit. A fixed concentration of protein (40 μg) was separated using 10% SDS-PAGE gel and transferred to a poly(vinylidene fluoride) membrane. The membrane was blocked with 5% skim milk powder in phosphate-buffered saline solution with 0.1% Tween 20 (PBST) at room temperature for 1 h, and later, the membrane was incubated at 4 °C overnight with the required primary antibodies PD-L1 (Genetex, Texas), Xct (Cell signaling, MA), HLA-A/B/C (MHC-I) (Asia bioscience, Taipei, Taiwan), Fas (Cell signaling), IL-10 (Genetex), Caspase 3 (Genetex) IFNγ (Cell signaling), Antiferritin (Abcam, Cambridge, U.K.), Glutathione Peroxidase 4 (GPX4) (Gentex), and GAPDH (Genetex). GAPDH was used as an internal control. After incubation, the membrane was washed three times with PBST for 15 min and then incubated with the respective secondary antibodies for 1 h. Subsequently, the membrane was washed four times with PBST for 10 min, visualized by enhanced chemiluminescence, and later detected in an AutoImager system (Amersham Imager 680, GE Healthcare and Bioscience, Princeton, NJ).

### Lipid Peroxidation Assay

Lipid ROS analysis was conducted using a Lipid Peroxidation Assay Kit (Asia Bioscience Co., Ltd., Taipei, Taiwan) designed to quantify the presence of malondialdehyde (MDA) (ab118970) as a marker of lipid peroxidation. For the analysis, A549 cells were seeded at a density of 1 × 10^6^ cells per well in a 6-well plate and incubated under conditions of 37 °C and 10% CO_2_ for 24 h. After 24 h, the cells were treated with miRNA34a and cocultured with inactive or active Jurkat T cells with and without miRNA34a. After 48 h of incubation, A549 cells were collected using the trypsin method and homogenized on ice with a homogenizer. The homogenized samples were subsequently subjected to centrifugation at 13,000*g* for 10 min. The resulting supernatant was collected and combined with a TBA (thiobarbituric acid) reagent, followed by an incubation period at 95 °C for 60 min. Finally, the optical density of the resulting solution was determined at a wavelength of 532 nm by utilizing a microplate reader.

## Results and Discussion

### Inactive Jurkat T Cells Inhibit the Cancer Cells in a Coculture

**Figure 2:**
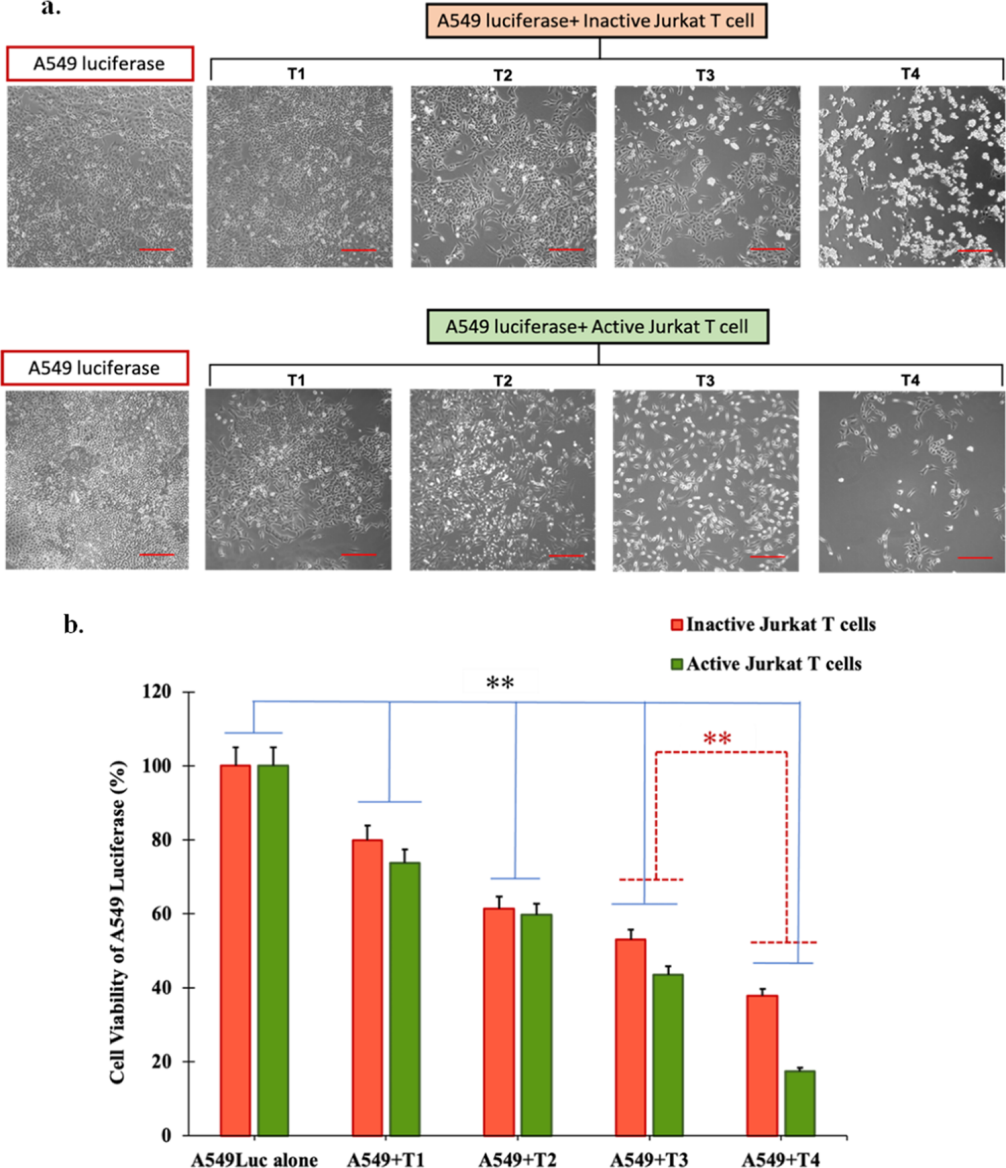
Coculture of A549 luciferase with activated or inactivated Jurkat T cells. (a) Microscopic image of A549 luciferase cells cocultured with an increasing number of inactive or active Jurkat T cells categorized as T1 (5 × 10^2^), T2 (5 × 10^3^), T3 (5 × 10^4^), and T4 (5 × 10^5^) (scale bar: 10 μM). (b) Luciferase assay was performed to measure the cell viability directly through luciferase activity in A549 luciferase cell lines after coculturing with inactive or active Jurkat T cells as compared to A549 cells alone (*n* = 3, *p < 0.05, **p < 0.01).

The cytotoxic potential of inactive or active Jurkat T cells was investigated by coculturing them with a fixed cell number of 1 × 10^5^ A549 luciferase lung cancer cells while varying the number of Jurkat T cells ranging from 5 × 10^2^ (T1) to 5 × 10^5^ (T4). Inactive Jurkat T cells were activated using the TransAct anti-CD3/CD28 reagent. Surprisingly, when observed under the microscope, the results demonstrate a decrease in the number of A549 cells with an increasing number of T cells (Figure 2). On the other hand, the population of inactive or active Jurkat T cells was still increasing, which indicates that the activity in Jurkat T cells was still there to attack the cancer cells, which can be seen in Figure 2a. The activity depicted by the inactive or active Jurkat T cells can be attributed to several factors and mechanisms. Jurkat T cells, especially when activated, possess cytotoxic capabilities and can induce cell death in target cells, including cancer cells like A549 cells.^29^ Upon coculture, Jurkat T cells can recognize and engage with A549 cells, leading to the release of cytotoxic molecules such as perforin and granzymes or the engagement of death receptors on A549 cells through ligand–receptor interactions.^31^ These cytotoxic mechanisms can result in the induction of apoptosis or cell death in A549 cells, leading to a decrease in their number.^32^ To confirm this microscopic visual evidence, luciferase assay was carried out to check the A549 luciferase cell activity in the coculture system varying with different numbers of T cells. The luciferase activity was measured as a proxy for the viability and metabolic activity of A549 luciferase cells. The results of the assay demonstrate a significant decrease in luciferase activity as the number of Jurkat T cells at 5 × 10^4^ in the coculture (Figure 2b). This finding suggests that even in their resting or inactive state, Jurkat T cells possess the ability to induce cell death in A549 cells. The enhancement of cell death in A549 cells is significantly dependent on the number of T cells. In comparison to the inactive Jurkat T cells, the luciferase activities of A549 cells show a significant decrease in the coculture with active Jurkat T cells at the numbers larger than 5 × 10^4^.

The observed cytotoxicity could be due to the inactive Jurkat T cells still expressing cell surface receptors capable of recognizing and interacting with ligands on the A549 cells.^32^ This interaction may trigger cytotoxic signaling pathways to release cytotoxic molecules such as perforin and granzymes, as shown in the Western blot analysis (Figure 4), which can induce apoptosis or other forms of cell death in the target cells.^29^

Apart from the above-mentioned possibility of T cells cytotoxicity, we also investigated the potential impact of coculturing A549 cells with Jurkat T cells on the expression of major histocompatibility complex class I (MHC-I) markers on the surface of A549 cells. MHC-I molecules play a crucial role in antigen presentation, enabling the immune system to recognize and eliminate abnormal or infected cells.^33^ IFN-γ also plays a crucial role in immunotherapy, particularly in enhancing the antitumor immune response. IFN-γ is a cytokine produced primarily by active T cells and natural killer cells in response to various stimuli, including interactions with cancer cells.^34^ These findings have important implications for understanding the complex interplay between immune cells and cancer cells. While the activation status of Jurkat T cells has traditionally been considered crucial for their cytotoxic function, this study highlights the inherent cytotoxic potential of inactive Jurkat T cells, emphasizing their significance as a model system for investigating T cell-mediated cytotoxicity.

### Jurkat T Cells Activated Using Anti-CD3/CD28

To enhance the activation and promote robust immune responses, Jurkat T cells were stimulated using T cell TransAct anti-CD3/CD28 reagent.^35^ Anti-CD3 antibodies bind to the CD3 complex on the T cell receptor (TCR), mimicking antigen recognition and providing the primary activation signal. Anti-CD28 antibodies interact with CD28 coreceptors on T cells, delivering a secondary costimulatory signal necessary for full T cell activation.^35^ We confirmed the activation of Jurkat T cells by conjugating the cells with CD3/CD28 primary antibodies and fluorescence-labeled secondary antibodies.

**Figure 3:**
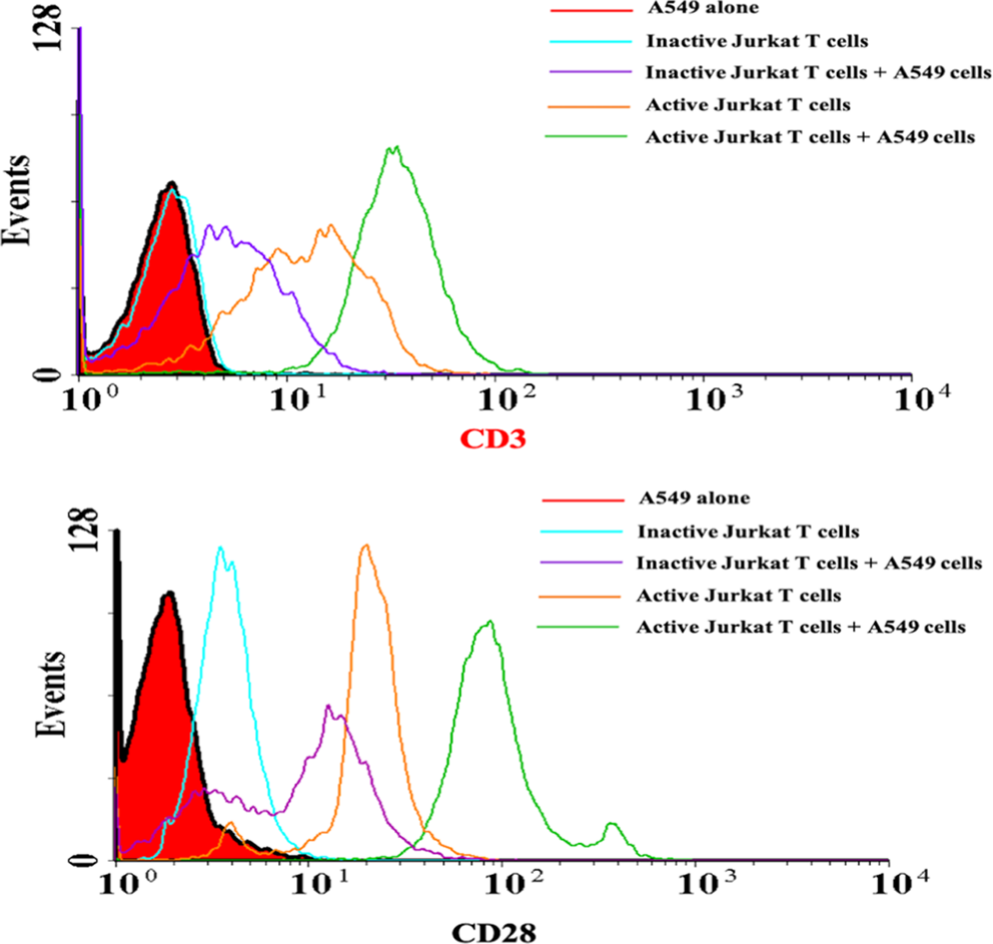
Flow cytometry analysis of Jurkat T cell activation markers CD3/CD28. Jurkat T cell activity before and after the stimulation with Transact anti-CD3/CD28 reagent and coculturing with A549 cells. The CD3/CD28 expression of inactivated Jurkat T cells increases after coculturing with the cancer cells, but after stimulation with anti-CD3/CD28, the expression of CD3/CD28 increases more significantly in coculturing the activated Jurkat T cells with A549 cells.

Flow cytometry analysis was conducted by incubating the inactive or active Jurkat T cells with CD3 and CD28 antibodies before and after coculturing with A549 cells. The results in Figure 3 reveal interesting findings regarding the expression of CD3 and CD28 on Jurkat T cells. The expression of CD3 and CD28 is significantly higher in the active Jurkat T cells compared to the inactive ones, indicating that the anti-CD3/CD28 activation successfully upregulates the expression of these important T cell markers.^36^

Furthermore, when the inactive Jurkat T cells were cocultured with A549 cells, there was an enhancement in the CD3/CD28 activity, as evidenced by increased CD3 and CD28 expression. This suggests that the presence of A549 cells stimulated the activity of inactive Jurkat T cells, possibly through interactions between cell surface molecules or the release of certain soluble factors.^29^ In contrast, when the active Jurkat T cells were cocultured with A549 cells, an even greater boost in CD3 and CD28 expression was observed. This implies that the active Jurkat T cells, already primed for activation, were further stimulated by the presence of A549 cells, resulting in a more pronounced increase in CD3 and CD28 expression, leading to an immune attack against the tumor cells.^3^ The interaction between A549 cells and Jurkat T cells, either through direct cell-to-cell contact or through the release of soluble factors, triggers signaling pathways that enhance CD3 and CD28 expression.^3^ Additionally, the engagement of T cell receptors with MHC molecules on A549 cells may further stimulate CD3/CD28 activity.^37^

### MiRNA34a Transfection in A549 Cells

The effects of miRNA34a transfection in A549 and Jurkat T cells coculture were analyzed by studying several oncogenic and tumor suppressor protein markers along with apoptosis, ferroptosis, and autophagy pathways. A549 cells (7 × 10^4^) were seeded, and post 24 h, 3 μg of miRNA34a was transfected and cocultured with 5 × 10^3^ inactive or active Jurkat T cells. To deliver miRNA34a into the A549 cells, IONRs were used as a gene delivery vector, and following the successful transfection along with the coculture with inactive or active Jurkat T cells, the cells were harvested post 48 h, and the Western blot analysis was employed to assess the expression levels of various signaling pathways including those that suppress or induce cancers by apoptosis, ferroptosis, or autophagy mechanisms.

**Figure 4:**
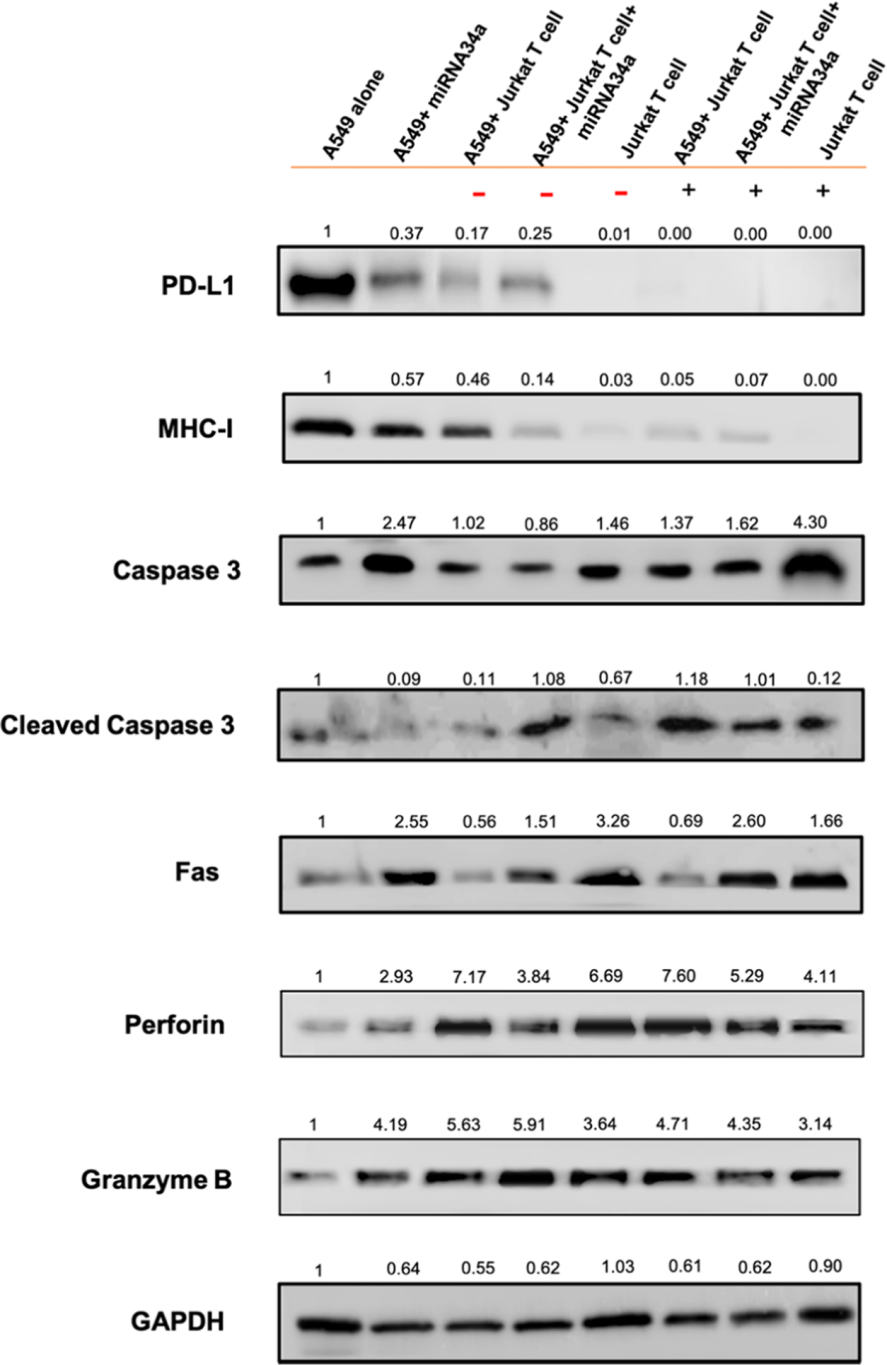
Western blotting analysis for immunotherapy checkpoint markers. After A549 cells were transfected with miRNA34a and cocultured with inactive Jurkat T cell (−) or active Jurkat T cell (+) cells, a successful knockdown in PD-L1 protein awakens the immune cells. Fas and MHC-I are present on cancer cell surfaces, showing higher knockdown after A549 cells are treated with miRNA34a and cocultured with inactive or active Jurkat T cells. The apoptotic Caspase 3 and cleaved Caspase 3 are upregulated, indicating the apoptosis activity in cancer cells. The cytotoxic molecules perforin and granzyme B also show higher expression in Jurkat T cells in their inactive and active states. GAPDH is used as an internal control for all of the other proteins. The protein expressions are calculated using a fold change method by using ImageJ software.

The expression of PD-L1, a ligand involved in immune checkpoint regulation, was evaluated to determine any changes in its levels after miRNA34a transfection and coculture with inactive or active Jurkat T cells (Figure 4). The transfection of miRNA34a in A549 cells results in a visible reduction in PD-L1 expression, indicating its potential role in downregulating PD-L1 levels. However, when the transfected A549 cells were subsequently cocultured with inactive (−) or active (+) Jurkat T cells, a comparative reduction in PD-L1 expression was seen. The samples with active Jurkat T cells show a dramatic decrease in PD-L1 levels as compared to the ones with inactive Jurkat T cells. The expression levels of PD-L1 reduce even more after A549 cells were transfected with miRNA34a and cocultured with both the inactive and active Jurkat T cells. The possible reasons for this enhanced reduction include the release of suppressive factors cytokines, such as interferons or tumor necrosis factor-α (TNF-α) by Jurkat T cells, activation of immune signaling pathways, and direct interaction between PD-1 on Jurkat T cells and PD-L1 on A549 cells.^38^ The combination of miRNA34a transfection and subsequent coculture with inactive or active Jurkat T cells results in a synergistic effect on enhanced reduction of PD-L1 expression in A549 cells.^39^

Similarly, the expression of MHC-I, which plays a crucial role in antigen presentation, was assessed to examine the alterations in its levels. The results in Figure 4 demonstrate a comparatively significant suppression of MHC-I expression in A549 cells following miRNA34a transfection. MiRNA34a is known to target multiple genes involved in immune regulation, such as PD-L1, and its transfection in A549 cells may directly affect the expression of MHC-I-related genes.^40^ As aforementioned, the PD-L1 expression on A549 cells reduced after miRNA34a transfection, and coculturing the transfected A549 cells with inactive or active Jurkat T cells further led to the suppression of MHC-I expression.^41^ The decrease in MHC-I expression could be attributed to the interaction between the Jurkat T cells and A549 cells during coculture, which might involve the release of soluble factors or cell-to-cell contact-mediated signaling that further downregulates MHC-I expression.^33^ The decreased PD-L1 expression on A549 cells after transfection with miRNA34a boosts the immune activity of cytotoxic T cells to recognize cancer cells and suppress their activity.^21^ For these reasons, we observed a higher suppression in the MHC-I expression when the cells were cocultured with active Jurkat T cells than those with inactive Jurkat T cells.

The Fas receptor, also known as CD95 or APO-1, is a cell surface receptor protein that plays a crucial role in apoptosis after binding with FasL, a ligand present on CD8+ T cells and also expressed by certain types of tumor cells.^42^ Upon recognition of cancer cells, active CD8+ T cells express and present FasL on their cell surface, which interacts with Fas, a death receptor protein present in the cancer cells, which leads to apoptosis in cancer cells.^43^ The cancer cells show resistance to Fas-induced apoptosis due to the lack of Fas receptors on the cell surface.^44^ Considering the apoptosis activity of the Fas/FasL pathway, the expression levels of Fas were also investigated. When miRNA34a-transfected A549 cells were cocultured with both inactive or active Jurkat T cells, an increase in Fas protein levels was observed in Western blot analysis (Figure 4). The expression of Fas is higher in the cells treated with miRNA34a with or without the presence of inactive or active Jurkat T cells. While the expression of Fas is not much enhanced when the A549 cells were cocultured with inactive or active Jurkat T cells, a slight increase was observed in the treatment of active Jurkat T cells as compared to the treatment of inactive ones. This could be related to the increased expression of PD-1 upon the proliferation of Jurkat T cells.^45^ MiRNA34a is often downregulated in various cancer types, and its reduced expression is associated with increased cancer cell survival by resisting apoptosis.^46^ Reintroducing miRNA34a into cancer cells or using miRNA34a mimics in cancer therapy has been explored as a strategy to inhibit tumor growth by promoting apoptosis.^47^ The binding of Fas and FasL triggers a series of intracellular signaling events, ultimately leading to the activation of caspases and resulting in the cleavage of key cellular proteins, DNA fragmentation, cellular membrane blebbing, and the formation of apoptotic bodies.^28^ The treatment of A549 cells with miRNA34a and active Jurkat T cells shows a much higher expression of the Fas protein than those with inactive Jurkat T cells. Thus, the synergy between the cells treated with miRNA34a and active Jurkat T cells proves to be an efficient immunotherapy strategy to kill cancer cells. The Fas-FasL pathway thus plays a crucial role in facilitating the cytotoxic activity of miRNA34a and Jurkat T cells against cancer cells and highlights its potential as a therapeutic target in cancer immunotherapy strategies.

The apoptosis pathway induction was further supported by the expression of Caspase 3 and cleaved Caspase 3 in the A549 cells transfected with miRNA34a and cocultured with inactive or active Jurkat T cells (Figure 4). MiRNA34a has been reported to target and suppress the expression of antiapoptotic proteins, such as Bcl-2, thereby favoring the activation of Caspase 3 and subsequent apoptosis induction.^48^ Thus, the upregulation of Caspase 3 and cleaved Caspase 3 levels after miRNA34a transfection can be attributed to the relief of its inhibitory regulation by Bcl-2.^49^ Further, the miRNA34a-transfected A549 cells cocultured with Jurkat T cells, even in their inactive state, could lead to the release of pro-apoptotic factors and cytokines, such as TNF-α and IFN-γ, which can further stimulate Caspase 3 activation.^50^ Altogether, the combined effects of miRNA34a transfection and coculturing with both inactive and active Jurkat T cells synergistically upregulate Caspase 3 and cleaved Caspase 3 levels in A549 cells, leading to enhanced apoptotic signaling and the potential elimination of cancer cells. Besides, the Caspase 3 activity was seen to be much higher in active Jurkat T cells as caspases have been found to be involved in T lymphocyte activation, and if the T cells are produced in excess, the caspases also lead to cell death.^51^ Caspase 3 is responsible for activation-induced cell death, which is processed once T cells are activated in the absence of apoptosis.^51^ Thus, the expression of Caspase 3 in the active Jurkat T cells indicates their proliferative behavior in the tumor microenvironment.

### Regulation of Ferroptosis Signaling Pathways

**Figure 5:**
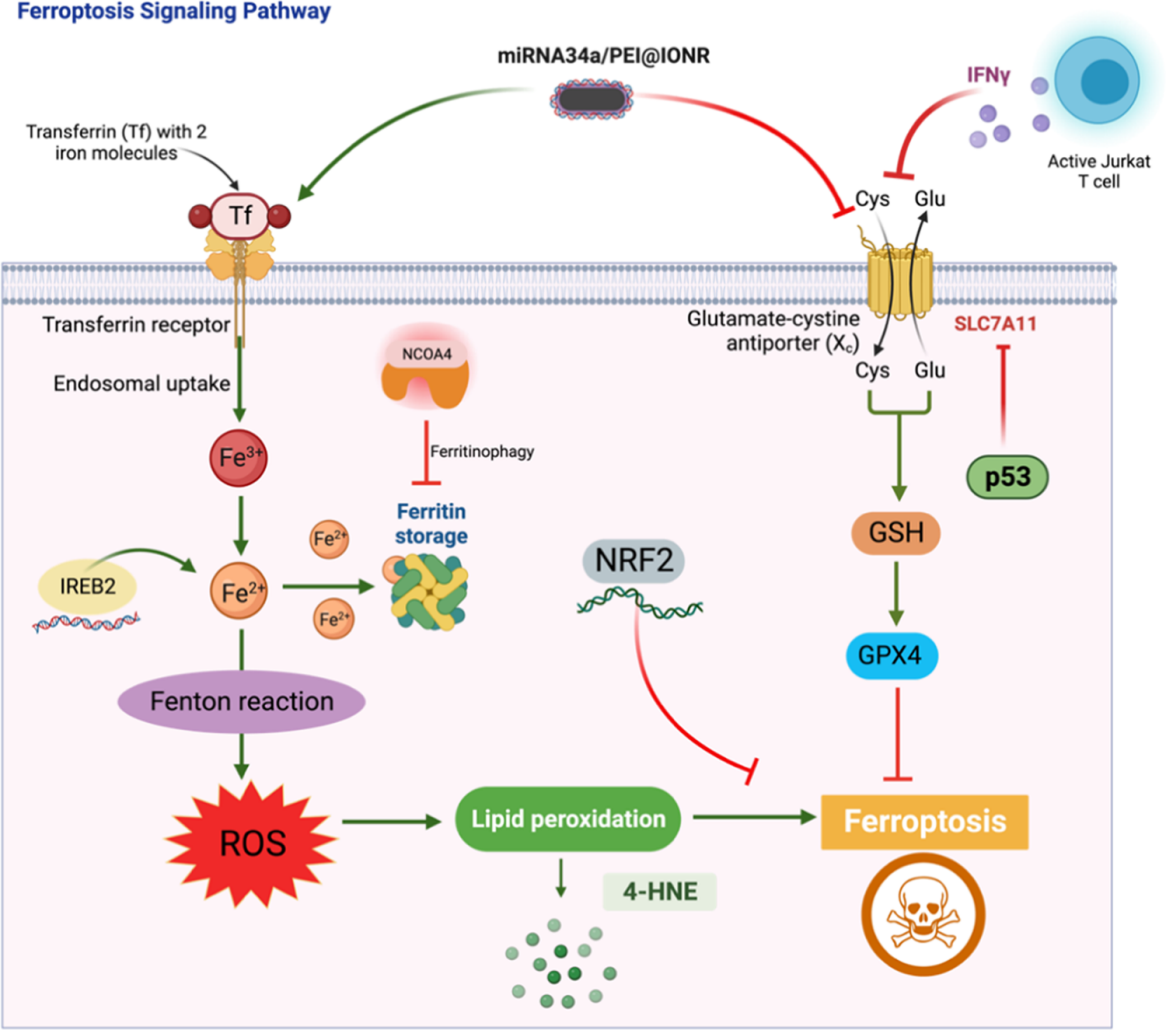
Schematic diagram of the ferroptosis signaling pathway. Many factors are found to be involved in lipid peroxidation synthesis as well as its inhibition. Iron is a central player in ferroptosis, and an excess amount of it can catalyze the formation of reactive oxygen species (ROS) through Fenton reactions, leading to cell membrane damage and lipid peroxidation, releasing 4-HNE as its byproduct. The cystine/glutamate antiporter SLC7A11/Xct is responsible for importing cystine, a precursor of glutathione synthesis. Enhancing SLC7A11/Xct activity can boost intracellular glutathione (GSH) levels, and GSH is a key antioxidant that helps protect cells from oxidative damage. The increase of intracellular glutathione levels activates GPX4, which is a critical factor in preventing ferroptotic cell death. Several genes and proteins, such as p53, IREB2, NRF2, and keap1, can regulate ferroptosis by modulating the expression of key enzymes and transporters involved in iron homeostasis and oxidative stress responses. Autophagy can help mitigate oxidative stress, which is a key driver of ferroptosis. Autophagy can selectively target and degrade ferritin, known as ferritinophagy, and can release iron into the cytoplasm.

Ferroptosis is a recently discovered form of regulated cell death that stands out for its unique biochemical and morphological characteristics.^52^ Unlike the well-known processes of apoptosis, necrosis, or autophagy, ferroptosis is distinct in its dependency on iron and lipid peroxidation.^53^ This intriguing cell death mechanism has garnered significant attention from researchers in cell biology, oncology, and neurodegenerative diseases due to its potential implications for various pathological conditions and its role in cellular homeostasis. We have investigated the fundamental aspects of ferroptosis by shedding light on its underlying mechanisms, as shown in Figure 5. There are several ways by which cancer cells can undergo ferroptosis. Here, we studied the role of miRNA34a and IONRs in ferroptosis and if the synergistic effect appears in the combination of miRNA34a and Jurkat T cells in cancer immunotherapy.

#### GSH and GPX Inhibition Required for Ferroptosis Activity

Glutathione (GSH) is a potent intracellular antioxidant in the cell that is key in protecting cells from oxidative stress by neutralizing harmful reactive oxygen species (ROS) and free radicals.^54^ Maintaining high levels of GSH is often essential for their survival and proliferation in cancer cells because it helps them combat the oxidative damage caused by their rapid growth and metabolism.^55^

**Figure 6:**
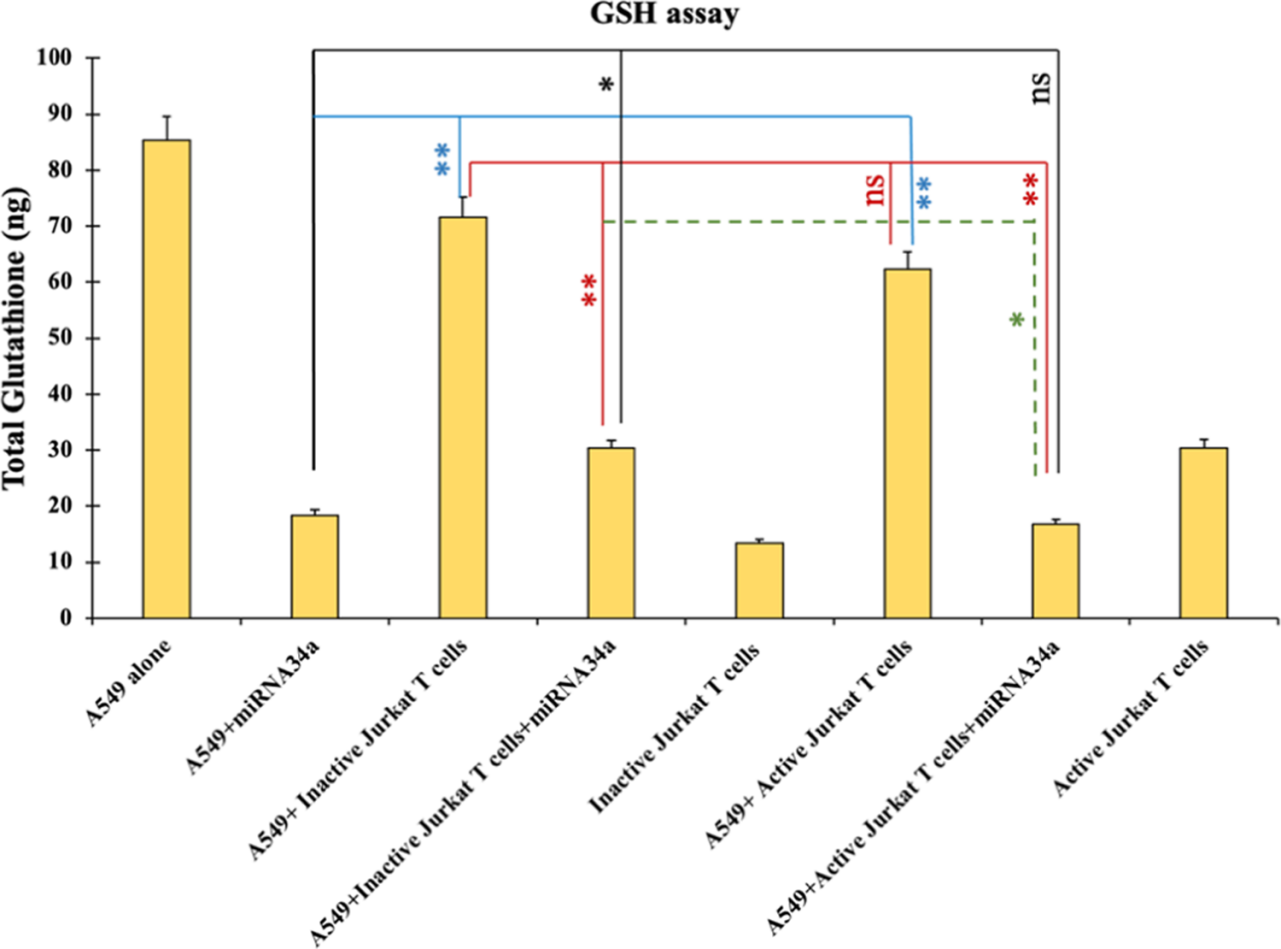
Glutathione Peroxidase Assay. The GSH assay was performed using a GSH kit, and the total GSH was measured in A549 cells treated with miRNA34a and cocultured with inactive or active Jurkat T cells and simultaneous treatment with miRNA34a in a coculture system. The total GSH shows a lower concentration in the cells treated with miRNA34a and in the coculture system treated with active or inactive Jurkat T after simultaneous treatment with miRNA34a. The comparison was made between miRNA34a-treated A549 cells vs coculture with inactive or active Jurkat T cells (*); miRNA34a-treated A549 cells alone vs coculture of A549 cells with inactive vs active Jurkat T cells alone (*); inactive Jurkat T cells and active Jurkat T cells with and without miRNA34a treatment (*); inactive Jurkat T cells or active Jurkat T cells treated with miRNA34a (*) (*n* = 3, *p < 0.05, **p < 0.01).

Cancer cells often produce elevated levels of ROS due to their increased metabolic activity and mitochondrial dysfunction. GSH helps protect these cells from oxidative damage, which can promote their survival. GSH is involved in the detoxification of harmful substances, including drugs and toxins. Cancer cells can also use GSH to detoxify chemotherapeutic agents, reducing the effectiveness of cancer treatments. Therefore, it is essential to inhibit the GSH synthesis, which can lead to ferroptosis in cancer cells.^56^ The depletion of GSH synthesis in cancer cells was observed when the cells were transfected with miRNA34a and cocultured with inactive or active Jurkat T cells. GSH assay was performed using a Glutathione Peroxidase Assay Kit in which total GSH was measured as per the manufacturer’s protocol. In Figure 6, a significant decrease in the GSH levels was seen in cancer cells treated with miRNA34a alone and with miRNA34a combined with inactive or active Jurkat T cells. This fact is because miRNA34a might have interrupted the upstream signaling pathways involved in ferroptosis inhibition, such as cystine-glutamate antiporter, also known as Xct,^57^ leading to the depletion of the downstream enzyme glutathione peroxidase 4 (GPX4).

**Figure 7:**
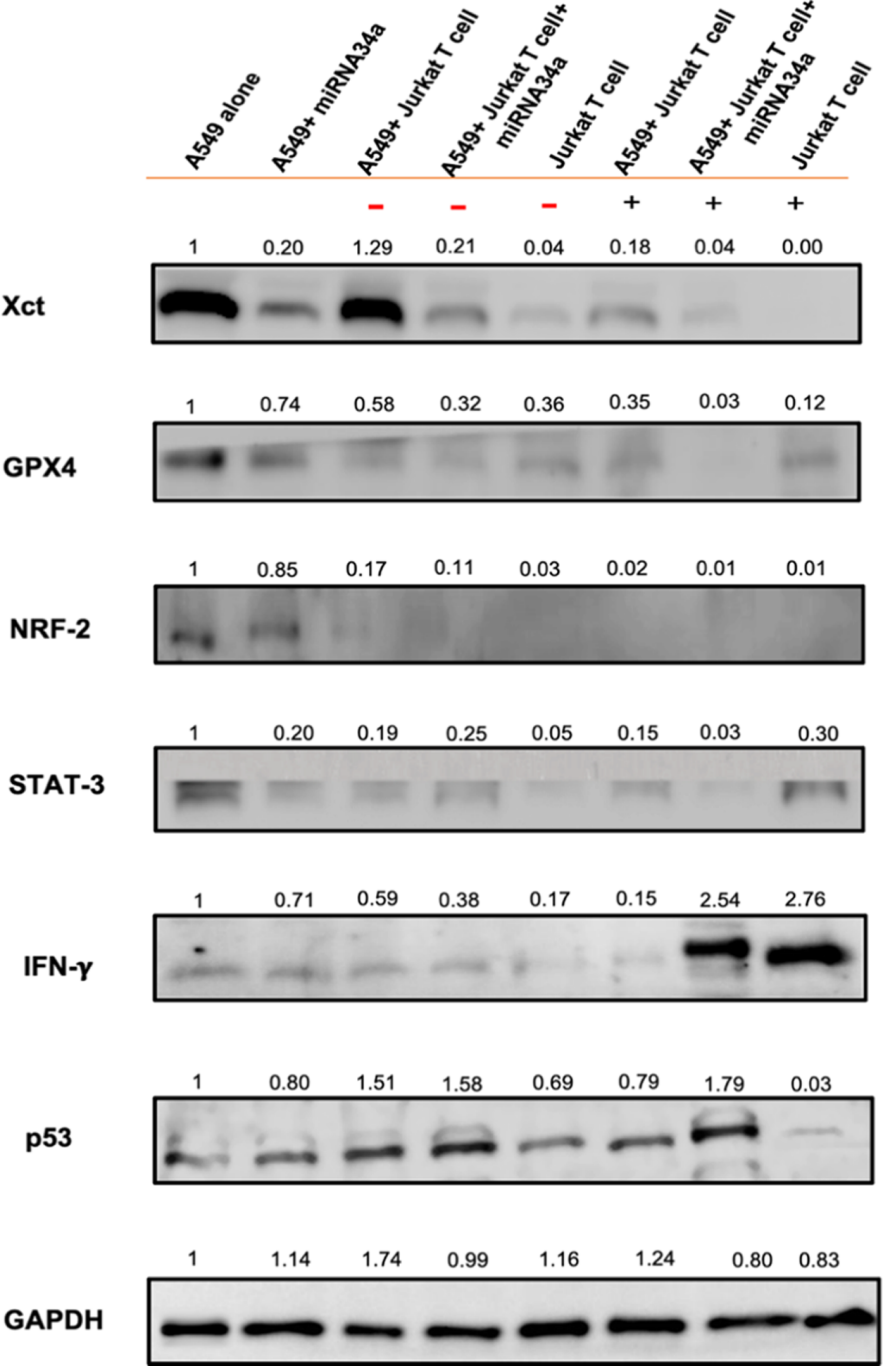
Western blotting analysis of ferroptosis signaling pathway. After A549 cells were transfected with miRNA34a and cocultured with inactive Jurkat T cell (−) or active Jurkat T cell (+) cells, a successful knockdown in SLC7A11, Xct, GPX4, and STAT3 is observed, thereby boosting the ferroptosis activity in cancer cells. IFN-γ and p53 show high expression in A549 cells transfected with miRNA34a and cocultured with active Jurkat T cells; GAPDH is used as an internal control for all of the other proteins. The protein expressions are calculated using the fold change method by using ImageJ software.

GPX4 is an enzyme that plays a crucial role in protecting cells against ferroptosis by reducing lipid hydroperoxides, including phospholipid hydroperoxides, and helps maintain cellular redox balance.^54^ In the synthesis of Glutamate–cysteine ligase, a limiting reaction leads to the formation of the glutamate–cysteine and then, GSH synthetase (GSS) provides for Glu-Cys link with glycine to obtain the tripeptide GSH.^58^ GSH is essential for the activity of GPX4 since it serves as a cofactor for GPX4 to reduce lipid hydroperoxides. In Figure 6, we have already seen a depletion in the levels of GSH in the cells treated with miRNA34a alone as well as a combinational treatment of miRNA34a in the presence of either inactive or active Jurkat T cells in A549 cells. Figure 7 shows the decrease in the GPX4 levels after A549 cells transfected with miRNA34a and cocultured with Jurkat T cells. In the context of ferroptosis, ferroptosis specifically targets and neutralizes lipid peroxides generated from the oxidation of polyunsaturated fatty acids (PUFAs) in cell membranes. When GPX4 activity is reduced, there is an accumulation of lipid peroxides in the cell membrane.^59^ In addition, there is a decrease in the GPX4 expression when the cells were cocultured with inactive or active Jurkat T cells without the transfection of miRNA34a. This situation might be due to T cells releasing IFN-γ, which in turn substantially reduced the expression of solute carrier family 7 member 11 (SLC7A11) and SLC3A2 in tumor cells.^60^ Consequently, the tumor cells experience diminished cystine absorption and increased lipid peroxidation and subsequently undergo ferroptosis.

SLC7A11 or Xct is a key component of the cystine/glutamate antiporter system that plays a crucial role in cellular antioxidant defenses and leads to ferroptosis in cancer cells.^61^ The primary function of the Xc-transporter is to import cystine,^57^ by providing cystine, which plays a crucial role in maintaining cellular redox balance and protecting cells from oxidative stress. Inhibition or downregulation of the SLC7A11/Xct leads to a decreased availability of cystine and subsequently reduced GSH synthesis, rendering cells more susceptible to lipid peroxidation and ferroptotic cell death.^62^Figure 7 shows the downregulation of SLC7A11/Xct protein levels after different treatments in A549 cells. The results suggest that miRNA34a could directly target and suppress the expression of SLC7A11/Xct mRNA, thereby leading to a decrease in SLC7A11/Xct protein levels and inducing ferroptosis death in the cancer cells.^63^ Interestingly, without the transfection of miRNA34a in A549 cells, the inactive Jurkat T cells show no such decrease in SLC7A11/Xct protein levels, but active Jurkat T cells show a dramatic decrease in their levels. This result is attributed to the inactive Jurkat T cells not producing enough IFN-γ factors or molecules directly affecting SLC7A11/Xct expression or activity, but when they were stimulated well by anti-CD3/CD28, sufficiently releasing IFN-γ enhances the suppression effect in SLC7A11/Xct expression. MiRNA34a can upregulate p53 activity by directly targeting inhibitors of p53 such as SIRT1 (silent mating type information regulation 2 homologue 1).^64^ The expression of SLC7A11/Xct can also be downregulated at the transcriptional level by p53. The repressed expression reduces the cystine uptake, and it is unavailability can limit glutathione synthesis and sensitize cells to ferroptosis.^65^

The nuclear factor erythroid 2-related factor 2 (Nrf2) is one of the crucial inhibitors of ferroptosis due to its ability to inhibit cellular iron uptake, limiting the production of ROS.^66^ On the other hand, SIRT1, known as sirtuin, a nicotinamide adenine nucleotide (NAD)-dependent protein deacetylase, is also involved in regulating the expression and activity of Nrf2.^67^ MiRNA34a is involved in the suppression of the Nrf2 by different mechanisms, such as by post-transcriptional suppression of SIRT1 protein expression and enhancing the p53 transcriptional activity and its stability.^68^ A549 cells show a low expression of Nrf2 in the cocultures with inactive or active Jurkat T cells and even lower expression in coculture cell systems with miRNA34a transfection (Figure 7). This high efficiency of the synergistic treatment in cancer cells holds a greater potential in the ferroptosis pathway and tumor suppression.

The potential of inactive or active Jurkat T cells was further confirmed by checking the levels of IFN-γ.^32^ Jurkat T cells are capable of producing and secreting IFN-γ upon activation, and their presence has significant implications for cancer cell killing.^1,69^ The Western blot image in Figure 7 shows a higher expression of IFN-γ when the cancer cells were cocultured with active Jurkat T cells as compared to the inactive ones. It stimulates the upregulation of cancer cell surface markers involved in immune recognition such as MHC-I and antigen-presenting markers. This facilitates the recognition and targeting of cancer cells by Jurkat T cells, ensuring efficient engagement and subsequent killing.^34^

#### Lipid Peroxidase and Iron-Mediated Ferroptosis

**Figure 8:**
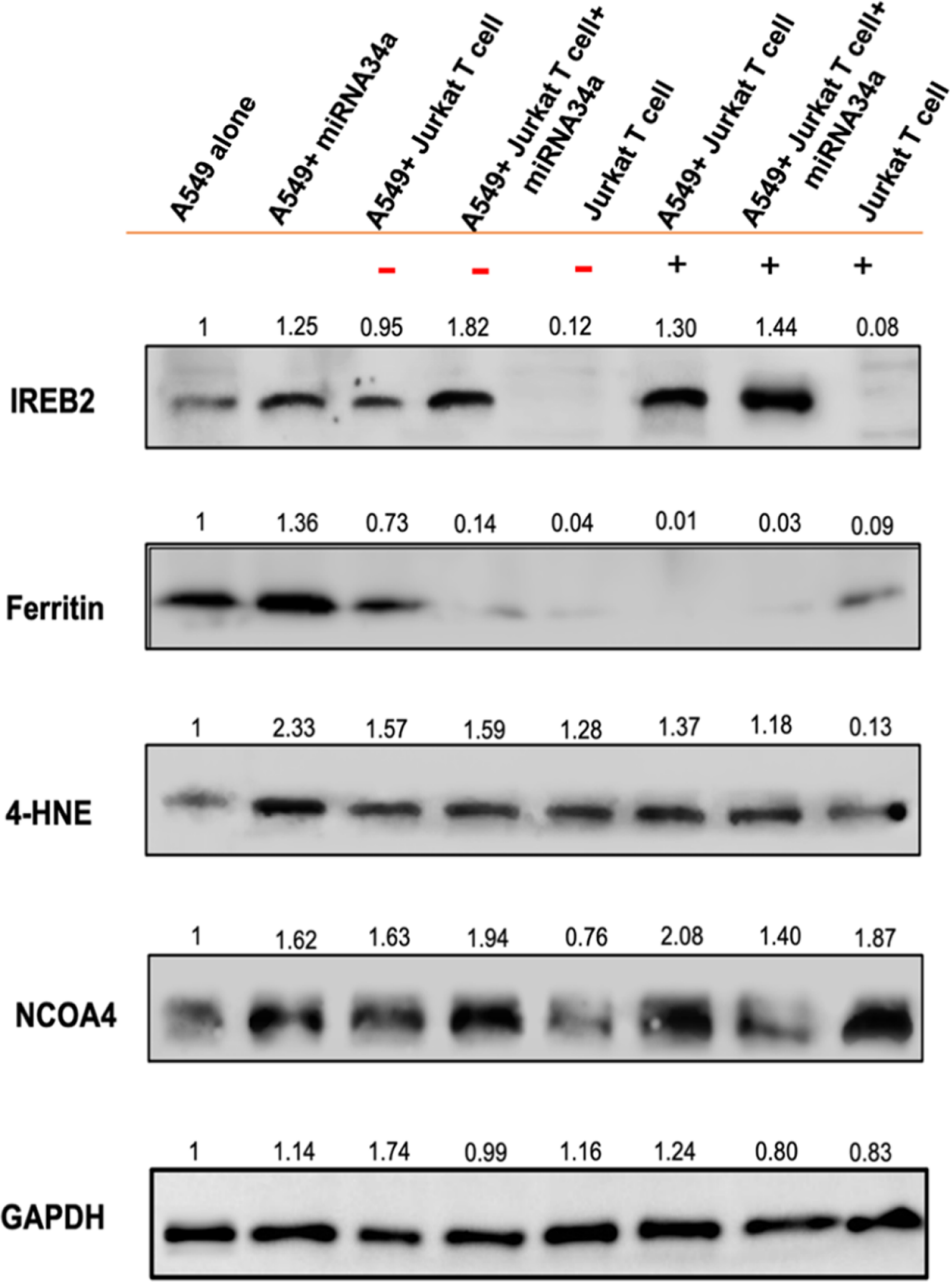
Western blotting analysis of ferroptosis signaling pathway. After A549 cells were transfected with miRNA34a and cocultured with inactive Jurkat T cell (−) or active Jurkat T cell (+) cells, a successful knockdown in Xct, GPX4 and STAT3 is observed, thereby boosting the ferroptosis activity in cancer cells. IFN-γ and p53 show high expression in A549 cells transfected with miRNA34a and cocultured with active Jurkat T cells; GAPDH is used as an internal control for all of the other proteins. The protein expressions are calculated using the fold change method by using ImageJ software.

We checked the ferritin level, which is a complex process influenced by various factors, including cellular iron levels, oncogenic signaling pathways, and post-transcriptional regulators such as microRNAs. We used IONRs to transfect miRNA34a, which can release iron ions within the cells, leading to an increase in intracellular iron levels. In response to the excess iron ions, cells may upregulate ferritin expression as a protective mechanism to sequester and store the surplus iron ions.^70^ In Figure 8, the Western blot image of ferritin showed an increase in levels after transfecting A549 cells using IONRs; however, the expression seems to be downregulated after coculture of the transfected A549 cancer cells with inactive or active Jurkat T cells. On the other hand, miRNAs can contribute to the suppression of ferritin function by binding to the mRNA of ferritin genes and inhibiting their translation. Several miRNAs have been identified as regulators of ferritin expression by targeting specific regions within the mRNA of ferritin genes (FTL and FTH1). One of the well-studied miRNAs in relation to ferritin regulation is miRNA-155.^71^ This post-transcriptional regulation of ferritin expression by miRNAs serves as a mechanism to control cellular iron levels and maintain iron homeostasis.^72^

Iron oxide nanoparticles have been studied for their potential role in cancer cells where they can generate ROS through Fenton-like reactions, which further can induce oxidative stress.^24^ The free Fe^3+^ released from IONRs binds to transferrin receptors, entering the cell through transferrin receptor 1 (TfR1), which is located near the endosome. Tf-TfR1 complex enters into endosome releasing iron from Tf, which is further transferred to the cytosol by divalent metal transporter 1 (DMT1) and combined with the labile iron pool (LIP).^73^ Iron plays a central role in ferroptosis by catalyzing the formation of highly reactive oxygen species (ROS) through Fenton and Haber–Weiss reactions. These reactions involve the interaction of iron ions with hydrogen peroxide (H_2_O_2_) to produce toxic hydroxyl radicals (^•^OH).^58^ Lipid peroxidation is the process by which ROS and free radicals attack and oxidize PUFAs within cell membranes.^60^ This process results in the formation of lipid hydroperoxides, which can be highly toxic to cells. As lipid peroxidation progresses, the cell membrane becomes leaky and loses its ability to maintain proper ion balance and cell structure, and such extensive damage to the cell membrane eventually leads to cell death.^54^ We explored the role of IONR-mediated miRNA34a transfection in ferroptosis as well as its impact on lipid peroxidation by investigating the iron-responsive element-binding protein 2 (IREB2) and 4-hydroxy-2-nonenal (4-HNE) expressions in the cells treated with miRNA34a alone and in combination with the inactive or active Jurkat T cells (Figure 8).

IREB2 plays a crucial role in regulating cellular iron homeostasis, and its involvement in ferroptosis is complex.^74^ In the controlled process of ferroptosis, this gene plays a significant role by binding to specific RNA structures called iron-responsive elements (IREs) found in the mRNA (mRNA) of genes involved in iron metabolism. Under conditions of iron deficiency, IREB2 binds to IREs on the mRNA of proteins involved in iron uptake, storage, and transport, preventing their degradation.^75^ This action promotes iron uptake and storage, helping increase intracellular iron levels to meet cellular needs. Increased IREB2 expression can indirectly enhance the iron-dependent processes involved in lipid peroxidation, which is a key feature of ferroptosis. The treatment of A549 cells with miRNA34a and synergistically coculturing with Jurkat T cell shows a significant increase in the expression of IREB2 compared to the untreated A549 cells. The expression seems to enhance more with the synergistic treatment with miRNA34a and active Jurkat T cells (Figure 8). The active Jurkat T cells help in immunosuppression of the cancer cells, thereby further increasing the ferroptosis effects on the cancer cells.

4-HNE is a well-known, highly reactive, and toxic lipid peroxidation byproduct that plays a significant role in ferroptosis.^60^ Lipid peroxidation involves the oxidative degradation of PUFAs within cell membranes. During this process, PUFAs are oxidized to form lipid hydroperoxides, including 4-HNE.^59^ This cycle of lipid peroxidation and 4-HNE formation contributes to increased oxidative stress within the cell, ultimately driving ferroptotic cell death.^76^ 4-HNE can inhibit the activity of downstream effectors GPX4, a key enzyme that detoxifies lipid hydroperoxides.^77^ GPX4 inhibition by 4-HNE reduces the cell’s ability to combat lipid peroxidation, facilitating the progression of ferroptosis. 4-HNE also showed an upregulation in the cells treated with miRNA34a and cocultured with inactive or active Jurkat T cells (Figure 8). This fact further contributed to the increase in oxidative stress after miRNA34a was transfected using IONRs with or without the synergistic effects of Jurkat T cells.

#### MiRNA34a and Jurkat T Cells Associated with Ferroptosis and Autophagy

An excess accumulation of Fe^2+^ will undergo oxidation to form Fe^3+^ ions with the help of a ferritin molecule.^66^ In abnormal functions of ferritin, if there is excess intracellular iron produced inside the cells, it is stored in ferritin. Therefore, it is important to suppress the activity of ferritin to avoid iron accumulation to stop hindering the process of lipid peroxidation to induce ferroptosis in cancer cells. Furthermore, to release the ferritin-bound iron for the use of cellular functions, nuclear receptor coactivator 4 (NCOA4), known for mediating ferritinophagy, results in iron release from ferritin, and the cellular labile pool is balanced.^78^ NCOA4 is a cargo receptor that recognizes and binds with the ferritin heavy chain (FTH1), which is one of the two subunits of the ferritin protein complex. This interaction is facilitated by a specific motif in NCOA4 known as the “LC3-interacting region” (LIR). NCOA4 acts as a bridge between ferritin and autophagosomes, which are double-membrane structures that are responsible for sequestering and delivering cellular cargo for degradation. Upon binding to ferritin, NCOA4 helps recruit the autophagosomal membrane to the ferritin-containing cellular compartment. This recruitment process leads to the formation of autophagosomes that envelop the ferritin-containing cargo, marking it for degradation.^79^

The reduced expressions of Ferritin and an increased expression of NCOA4 were clearly observed in A549 cells treated with the combination of miRNA34a and inactive or active Jurkat T cells (Figure 8). The NCOA4 expression is high in the cells treated with miRNA34a and cocultured with either inactive or active Jurkat T cells. MiRNA34a has been shown to be effective on autophagy-related genes (ATG), such as ATG4, ATG5, and ATG9.^80^ The interaction between T cells and autophagy in cancer is complex and context-dependent. IFN-γ, an important factor for the activation of cytotoxic T cells (CD8+), has been shown to induce autophagy of tumor cells with the process of autophagosome formation and maturation.^81^

Signal transducer and activator of Transcription 3 (STAT3) is an oncogene responsible for the transcription of various cellular processes, including cell survival, proliferation, and immune response. STAT3 can influence ferroptosis by modulating the expression of the antioxidant genes. It has been shown to upregulate the transcription of genes encoding antioxidants such as GPX4 and SLC7A11, which are crucial in protecting cells from lipid peroxidation. Increased STAT3 activity can enhance the expression of these protective genes, reducing the susceptibility of cells to ferroptosis.^82^ MiRNA34a targets the negative γ globulin regulator genes, including STAT3, thereby regulating the γ-globin gene expression inside the cells. When A549 cells were transfected with miRNA34a, a visible decrease in STAT3 expression was seen (Figure 7). The coculture of A549 cells with inactive or active Jurkat T cell also shows the suppression in the STAT3 protein, probably because IFN-γ can induce the expression of proteins like SOCS1 (suppressor of cytokine signaling 1) and PIAS3 (protein inhibitor of activated STAT3), which are negative regulators of STAT3 signaling.^83^ These proteins can bind to STAT3 and inhibit its transcriptional activity. However, the simultaneous treatment using miRNA34a and active Jurkat T cells shows a drastic decrease in the expression of the STAT3 protein. This result holds the strong potential of a synergistic approach to kill cancer cells.

**Figure 9:**
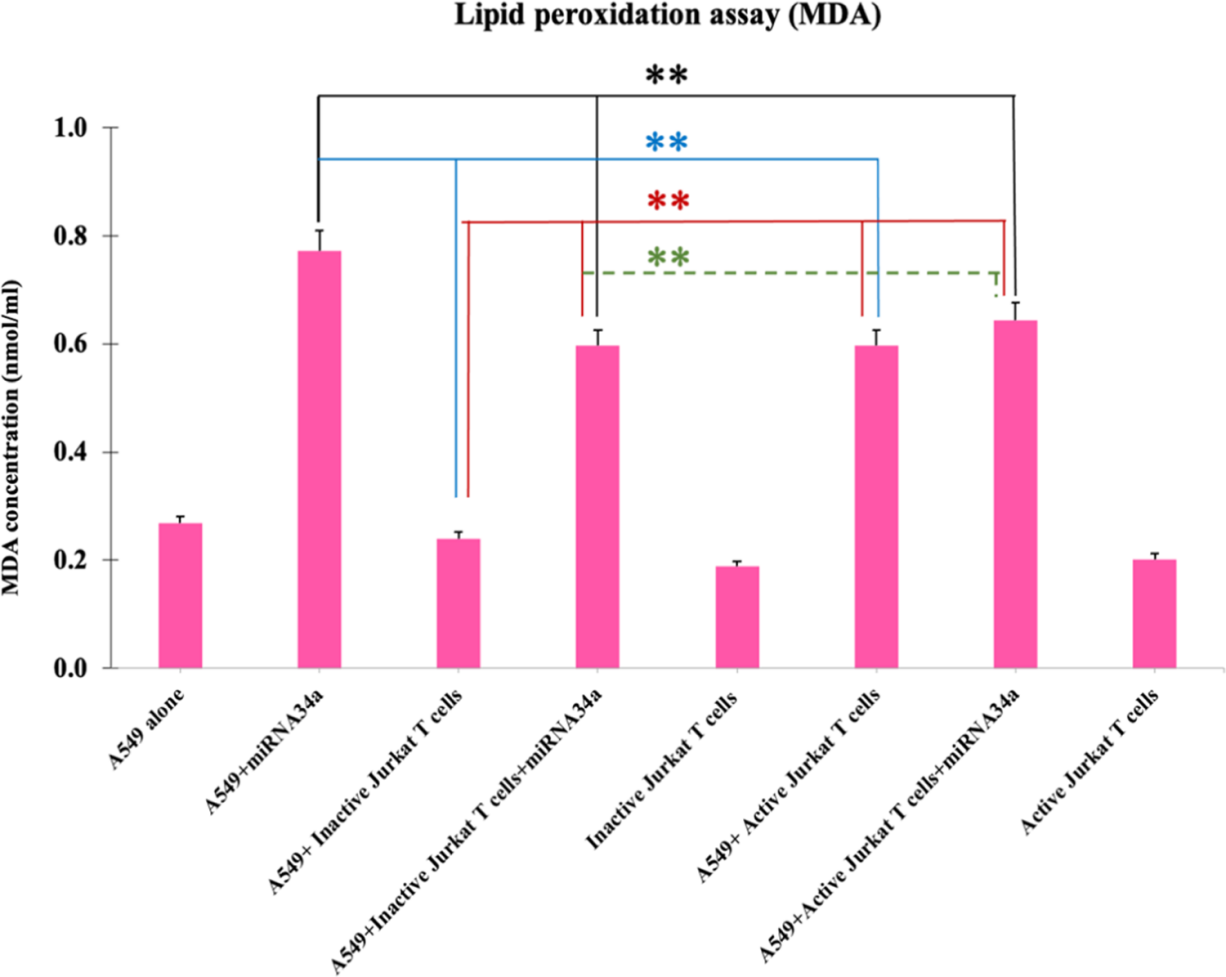
Lipid peroxidation assay. Lipid peroxidation assay was carried out after treating A549 cells with miRNA34a and coculturing with inactive or active Jurkat T cells and simultaneous treatment with miRNA34a in a coculture system. The MDA concentration was measured, and it showed a higher concentration in the cells treated with miRNA34a compared to the coculture with inactive or active Jurkat T cells with and without miRNA34a synergistic treatment. The comparison was made between miRNA34a-treated A549 cells vs coculture with inactive or active Jurkat T cells (*); miRNA34a-treated A549 cells alone vs coculture of A549 cells with inactive vs active Jurkat T cells alone (*); inactive Jurkat T cells or active Jurkat T cells with and without miRNA34a treatment (*); inactive Jurkat T cells treated or active Jurkat T cells treated with miRNA34a (*) (*n* = 3, *p < 0.05, **p < 0.01).

We also quantified the level of lipid peroxidation to measure the oxidative stress in cancer cells after treatment with miRNA34a and coculturing with inactive or active Jurkat T cells. Lipid peroxidation forms reactive aldehydes such as MDA and 4-HNE as natural byproducts and is commonly used as a marker of lipid peroxidation and to assess oxidative stress.^54^ The measurement of MDA was carried out using a Lipid Peroxidation Assay Kit to assess the oxidative stress levels within cells. The cancer cells were subjected to treatment with miRNA34A and cocultured with both active and inactive Jurkat T cells and further processed for MDA measurement after 48 h post-treatment. The analysis of lipid peroxidase levels in these cells reveals a notable increase in MDA concentration in the group treated with miRNA34a as well as the coculture group with miRNA34a and active Jurkat T cells (Figure 9). MiRNA34a has been reported to play a significant role in regulating oxidative stress by targeting key genes, such as Ferritin, Xct, and Nrf2, as observed in Western blot analysis (Figures 7 and 8), which are involved in antioxidant defense mechanisms. By inhibiting these genes, miRNA34a can promote the accumulation of ROS within cells, thereby enhancing lipid peroxidation.^63^

The presence of active Jurkat T cells likely contributes to the observed increase in lipid peroxidation. When T cells are activated, there is a rapid increase in glucose uptake and glycolysis for ATP production.^84^ Moreover, oxidative phosphorylation in T cells is increased, which is essential for ROS production as part of their immune response, aimed at eliminating foreign or abnormal cells such as cancer cells.^84^ This oxidative environment created by active T cells may synergize with miRNA34a-induced oxidative stress, leading to higher levels of lipid peroxidation.

### Apoptosis Activity of Jurkat T Cells on Cancer Cells

**Figure 10:**
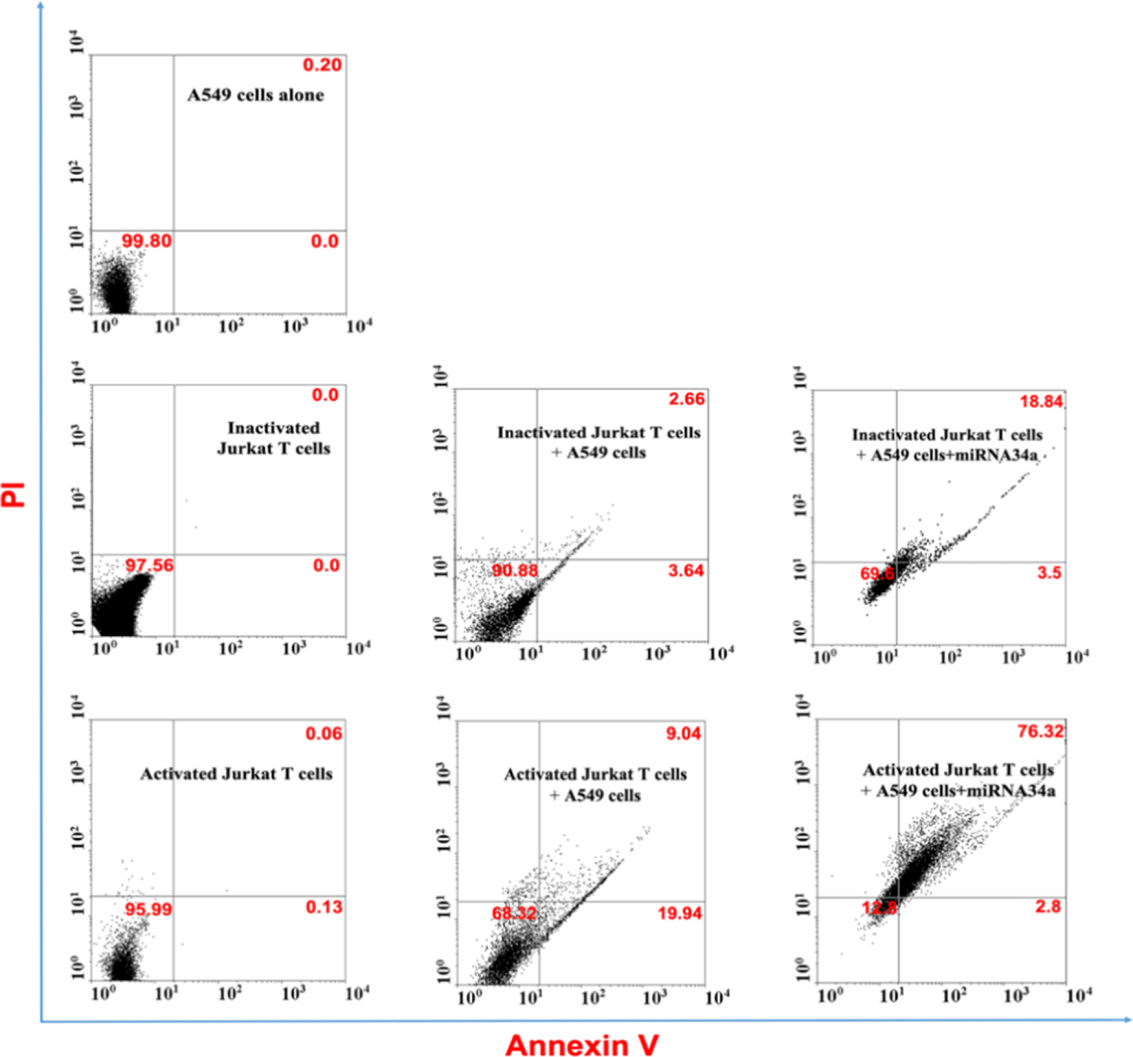
Flow cytometry analysis to study cell apoptosis. The apoptosis activity of A549 cells with and without miRNA34a transfection and coculture with active or inactive Jurkat T cells. The flow cytometry analysis indicates that miRNA34a-transfected A549 cells cocultured with active Jurkat T cells have the largest population of cells located in early and late apoptosis phases Q4 and Q2 (the lower right and upper right corner) as compared to the coculturing inactive Jurkat T cells among test groups. This indicates that activated Jurkat T cells develop more potential to kill cancer cells as compared to the inactive ones. The results are represented as mean value ± standard deviation (*n* = 3, *p < 0.05, **p < 0.01).

**Figure 11:**
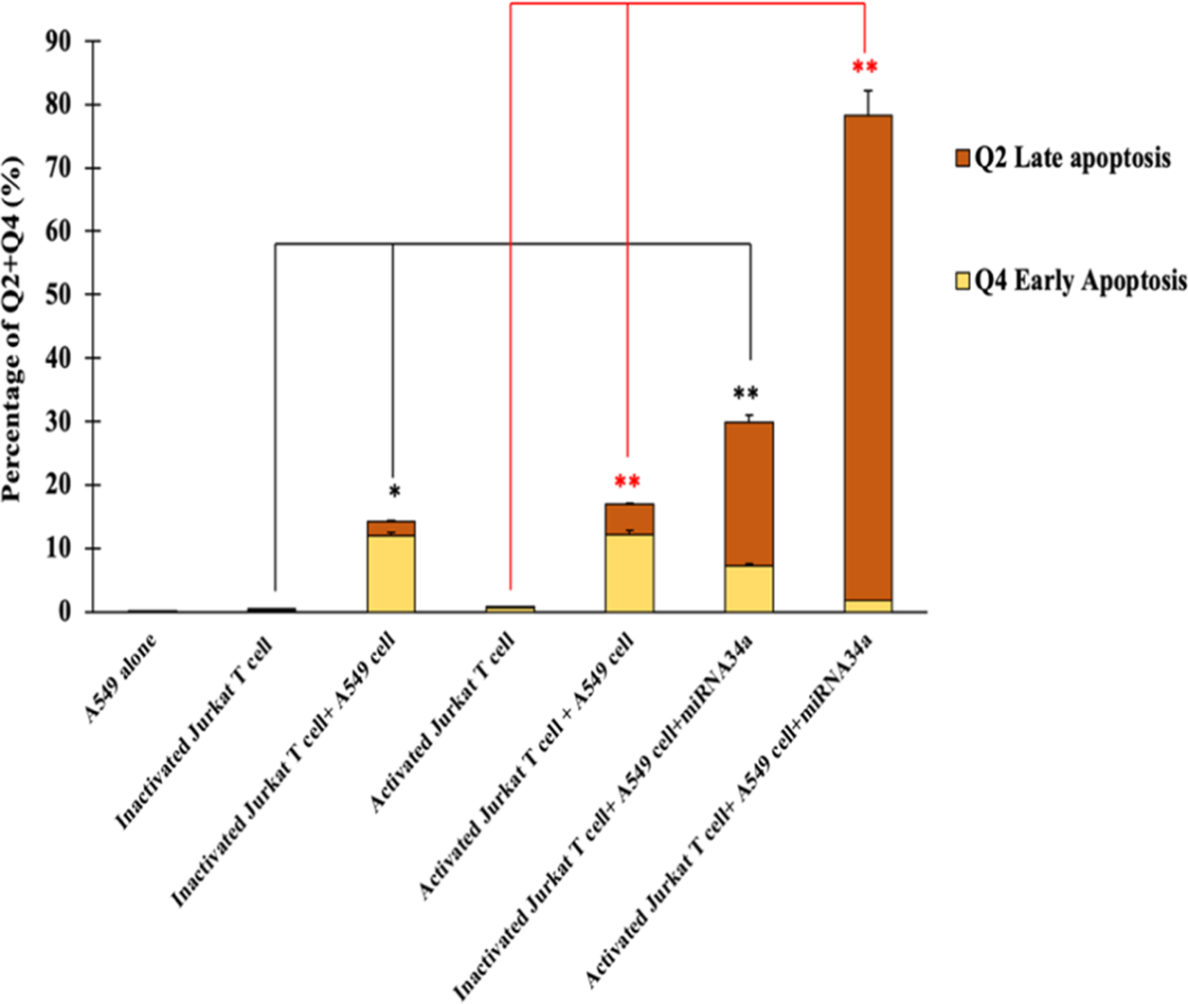
Quantification of flow cytometry percentage of early apoptosis and late apoptosis in active or inactive Jurkat T cells in a coculture system with and without miRNA34a in A549 cells. Inactive or active Jurkat T cells are used as a positive control to test the coculture treatments. The results are represented as mean value ± standard deviation (*n* = 3, *p < 0.05, **p < 0.01).

The apoptosis activity of A549 cells with and without miRNA34a transfection and coculture with active or inactive Jurkat T cells was further confirmed using the dual Annexin V/PI staining method. The coculture of A549 cells with Jurkat T cells boosted the cytotoxic efficiency of the A549 cells, even in an inactive state. In Figures 10 and 11, A549 cells, inactive Jurkat T cells, and active Jurkat T cells all show low apoptosis activity, but A549 cells cocultured with inactive Jurkat T cells show some extent of cell apoptosis. Comparatively, A549 cells cocultured with active Jurkat T cells show a higher rate of apoptosis. A visible difference in the apoptosis activity of A549 cells transfected with miRNA34a is seen in the coculture system between active and inactive Jurkat T cells. MiRNA34a functions as a tumor suppressor by targeting a wide range of genes involved in cell survival, proliferation, and antiapoptotic pathways.^21^ MiRNA34a indirectly modulates the expression of various antiapoptotic proteins, including B-cell lymphoma 2 (Bcl-2), myeloid cell leukemia sequence 1 (MCL-1), and survivin.^46^ MiRNA34a also participates in the regulation of multiple apoptotic signaling pathways. It has been shown to modulate the phosphatidylinositol 3-kinase/protein kinase B (PI3K/AKT) pathway, which is known for its antiapoptotic properties. MiRNA34a inhibits AKT activation, thus dampening the survival signals mediated by this pathway and sensitizing cells to apoptotic stimuli. The multifaceted functions of miRNA34a in apoptosis induction highlight its potential as a therapeutic target for various diseases, particularly cancer.^47^ Therefore, after transfecting A549 cells with miRNA34a and coculturing with active or inactive Jurkat T cells, the apoptosis activity increases significantly, as miRNA34a boosts the cancer cell killing mechanism via apoptosis. On the other hand, Jurkat T cells could also induce cancer cell death by releasing cytotoxic molecules, such as perforin and granzymes, or by inducing apoptosis by the Fas-FasL pathway, which is due to the engagement of death receptors on A549 cells by Jurkat T cell-expressed ligands.^43^ This synergistic approach by using miRNA34a and Jurkat T cells boosts the cancer cell killing mechanisms by inducing strong apoptosis activity, thereby making this one of the potential immunotherapeutic treatments in patients suffering from cancer.

### Discussion and Conclusions

Our research on immunotherapy involving the coculture of A549 cells with active or inactive Jurkat T cells provides valuable insights into the cytotoxic properties of T cells and their role in cancer cell killing. We found that in an inactive state, Jurkat T cells retain the ability to target and eliminate cancer cells, although to a lesser extent compared to their active counterparts. Western blot results likely revealed the presence of specific proteins associated with inactive T cell cytotoxicity, such as Fas and FasL, along with the activation of Caspase 3, indicating their involvement in inducing cancer cell death. The introduction of miRNA34a with inactive or active Jurkat T cells synergistically in A549 cells likely influenced ferroptosis through several mechanisms such as downregulation of antioxidant defenses by miRNA34a, which may downregulate the expression of antioxidant genes, reducing the cell’s ability to combat lipid peroxidation. Several key ferroptosis markers were studied, which are positively and negatively involved in the ferroptosis signaling pathway, such as GPX4, 4-HNE, Ferritin, p53, IFN-γ, Xct/SLCA711, etc. The necrosis phenomenon was also seen in this combined immunotherapy approach. The synergistic effects observed in this strategy, including the induction of apoptosis, ferroptosis, and necrotic cell death, highlight the potential for enhanced cancer treatment outcomes.

The synergistic antitumor effect observed when cytotoxic T cells and miRNA34a are combined on cancer cells is rooted in the complementary actions of these two powerful components, each targeting distinct aspects of cancer cell survival and proliferation. Cytotoxic T cells induce apoptosis in cancer cells through direct cytotoxicity by releasing factors like perforin, granzyme, and IFN-γ, while miRNA34a amplifies this effect by downregulating the PD-L1 gene, which promotes apoptosis through its regulatory actions on key apoptotic pathways. The combined action of cytotoxic T cells and miRNA34a results in an enhanced and coordinated apoptotic response in cancer cells. In addition, when IONRs are used as a delivery agent for miRNA34a to deliver inside tumor cells using a static magnet, IONRs allow for more targeted delivery of therapeutic agents to tumor cells, reducing potential damage to healthy tissues. While Jurkat T cells induce apoptosis, IONRs contribute by triggering ferroptosis. These nanoparticles can serve a dual purpose by not only inducing ferroptosis but also acting as contrast agents for imaging. This allows for the monitoring of treatment progress and the assessment of the distribution of therapeutic agents within the body. In summary, the synergistic antitumor effect of Jurkat T cells, miRNA34a, and iron oxide nanoparticles arises from their complementary mechanisms of action, addressing different aspects of tumor cell survival and evasion. The combination offers a potential strategy for enhanced therapeutic outcomes while minimizing the side effects. However, it is essential to note that the success of such a therapeutic approach would require careful optimization of dosages, delivery methods, and potential challenges associated with the immune response and nanoparticle toxicity.

As research in this field continues to evolve, the combination of T-cell-based immunotherapy and miRNA34a-based interventions holds great promise for the development of more effective and targeted cancer treatment modalities, offering new avenues for improving patient outcomes and advancing the fight against cancer.
